# Non-conventional humanitarian interventions on Ebola outbreak crisis in West Africa: health, ethics and legal implications

**DOI:** 10.1186/2049-9957-3-42

**Published:** 2014-11-25

**Authors:** Ernest Tambo

**Affiliations:** Sydney Brenner Institute for Molecular Bioscience, Wits 21st Century Institute, Faculty of Health Sciences, University of the Witwatersrand, Johannesburg, South Africa; Center for Sustainable Malaria Control, Department of Biochemistry, Faculty of Natural and Agricultural Sciences, University of Pretoria, Pretoria, South Africa; National Institute of Parasitic Diseases, Chinese Centre for Disease Control and Prevention, and the WHO Collaborating Centre on Malaria, Schistosomiasis and Filariasis, Shanghai, 200025 People’s Republic of China; Faculté des Sciences Biomédicales et Pharmaceutiques, Université des Montagnes, Bangangté, République du Cameroun

**Keywords:** Non-conventional, Response, Ethics, Legal, Ebola, Humanitarian crisis, Africa

## Abstract

Due to the lack of Ebola outbreak early warning alert, preparedness, surveillance and response systems, the most deadly, complex and largest ever seen Ebola war has been devastating West African communities. The unparalleled Ebola tsunami has prompted interrogations into, and uncertainties about, the effectiveness and efficiency of national, regional and international community’s illed- responses using conventional humanitarian control and containment approaches and methods. The late humanitarian and local non-government organisations emergency responses and challenges to curb transmission dynamics and stop the ongoing spread in the Ebola outbreak in West Africa have led to an unprecedented toll of 14,413 reported Ebola cases in eight countries since the outbreak began, with 5,177 reported deaths including 571 health-care workers and 325 died as 14 November 2014. These indications the need of further evaluation of monitoring as substantial proportion of infections outside the context of Ebola epicentres, Ebola health centres treatment and care, infection prevention and control quality assurance checks in these countries. At the same time, exhaustive efforts should target ensuring an sufficient supply of optimal personal protective equipment (PPE) to all Ebola treatment facilities, along with the provision of training and relevant guidelines to limit to the minimum possible level of risk. The continent hosts a big proportion of the world’s wealth, yet its people live in abject poverty, with governments unable to feed and govern them effectively, and who are condemned to endure even darker moments with the Ebola outbreak in West Africa. Institutionalisation of practical and operational non-conventional emergency response models efficient health systems, and tailored programmes can clearly support to prevent, control and eventually stamp out Ebola geo-distribution in addition to population mental health services that are requisite to address the massive range of the health, socio-psychological and economic consequences during and post Ebola associated crises. There is a critical need for a more pragmatic and robust scientific approach to transform and re-orient the huge natural and human resource potentials towards achieving universal coverage, the 2015–2030 Millennium Developing Goals (MDGs), sustainable growth and development in Africa.

**Electronic supplementary material:**

The online version of this article (doi:10.1186/2049-9957-3-42) contains supplementary material, which is available to authorized users.

## Multilingual abstracts

Please see Additional file 1 for translations of the abstract into the six official working languages of the United Nations.

## Introduction

### Review of tsunami scale humanitarian crisis in West Africa

The tsunami scale humanitarian crisis in West Africa Ebola outbreak is the largest, most complex and most severe ever seen. Compared to previous episodes in parts of Africa, this outbreak was underestimated. Humanitarian organisations have been besieged by the tenacious wave of new cases, which far outgrows the available basic medical, and health capacities and late emergency responses [1]. The potential Ebola pandemic and the negative impact thus far with conventional processes and tools deployed being largely unsuccessful underscore the urgent need for rapid rethinking and/or re-engineering of innovative approaches including the use of non-conventional intervention(NCI) methods and actions which are prohibited by international health regulations under emergency humanitarian crisis, but could be effective to prevent further Ebola spread, save the lives of millions and protect the regional and world’s economy. NCI in Ebola tragedy appears to be new and frightening may be due to the undertone and previous documented impact of such strategy in crisis control and management worldwide [1, 2]. However, should NCI effective in Ebola virus outbreak and humanitarian crisis prevention and containment in West Africa, offer a novel 21^st^ century approach and tools for target and timely emergency actions, partnership and empowerment of the communities, strengthening of rapid case identification and contact tracing, infrastructure development for patients care and effective quarantine of suspected and relatives contact with patients, patients body fluids or deceased, proper protection of healthcare workers, monitoring and evaluation (M&E). Such evidence is yet to be established through the ongoing NCI on Ebola widespread in West Africa, mainly in Liberia and Sierra Leone.

There are five subtypes of the Ebola Virus Disease (EVD), each named after its country of origin: *Ebola Zaire, Ebola Cote d’Ivoire, Ebola Sudan, Ebola Reston and Ebola Bundibugyo*. The disease is classified as a viral haemorrhagic fever. The EVD is introduced into the human population through close direct contact with the blood, organs or other body fluids of infected animals or people. Those who have had direct contact with bodily fluids of a person/patient who is infected with the Ebola virus, who have handled a body of a person who died of Ebola, healthcare workers working with patients infected with the Ebola virus, and family and friends of patients with Ebola are at a higher risk. Burial ceremonies in which mourners have direct contact with the body of the deceased person can also play a role in the transmission of the virus. Healthcare workers have been infected while treating patients with suspected or confirmed EVD, when infection control measures are deficient. The incubation period varies between 2–21 days and is, most often, less after exposure. It is characterised by severe fever, haemorrhaging (bleeding), multiple organ failure and often death [2].

The Ebola outbreak in West Africa is considered to be one of the world’s deadliest to date. The EVD illnesses are increasing exponentially in Liberia, where taxis are literal vehicles of disease transmission as they ferry sick people between treatment centres that are too full to admit them. At least 5,176 people have died of the virus in Guinea, Liberia, Sierra Leone, Nigeria and Mali, and the virus has resulted in more than 8,000 orphans [1, 2]. The situation is extremely worrisome, and health workers themselves are becoming scared of treating patients, which puts a further strain on the health services of the West African states that have historically faced a shortage of doctors, facilities and supplies, as well as poor health infrastructure and facilities [3]. Neighbouring countries including Ivory Coast and Senegal have shut their borders, and airlines are suspending flights to affected countries. More than 300 doctors and nurses have died in the Ebola epidemic in West Africa due to a lack of, and/or, challenges and limitations in implementation of appropriate conventional outbreak control and containment measures. Moreover, other reasons have been ascribed and contributed to the worsening the West Africa crisis such as: lack of timely international community action, uncoordinated humanitarian organisations, lack of emergency response models in limited resources settings, apprehension and resistance of local population, traditional setting culture and attitudes to health, and health seeking behaviour and attitudes, weak or inexistent epidemic preparedness, early alert systems and contingency plans, regional and global outbreak and emergency response unpreparedness, inadequate moral and psychological guide and counselling to, healthcare workers, domestic and foreign staff, and population, poor governance and corruption, inability and failure to meet up with the endorsed Abuja declaration in investing 15% of their national Gross Domestic Products (GDP) into Health since 2000, lack of accountability and transparency in most systems, dearth local resource staff shortages in addition to rural community health center and other personal protective equipment [3, 4]. Also, there are poor or no early warning alert and surveillance systems and evidence-based responses [5]. To compound matters, sending supplies and additional medical staff to help the affected communities has become more difficult due to flight cancellations and border closures which is aimed at averting the increasing spread of the Ebola infection in the region and preventing a global pandemic by curbing the ongoing transmission dynamics [1, 6]. The World Health Organisation’s (WHO’s) assessment, based on reports from an emergency team in Liberia, said that ‘non-conventional interventions’ are needed to control the outbreak as the demands have outstripped the government’s and partners’ capacity to respond effectively. The situation requires further support and mobilisation of innovative approach such as non-conventional intervention assets to stem out the persistent Ebola outbreak’s geo-distribution and dynamic spread across West Africa [7].

The Ebola outbreak humanitarian crisis is characterised by a series of events which represent a critical threat to the health, safety, security and wellbeing of the local, national and international community. This crisis may evolve and/or extend beyond the mandate or capacity of any single agency. Such humanitarian crises can be grouped under the following headings: (1) natural disasters (earthquakes, floods, storms and volcanic eruptions); (2) man-made disasters (conflicts, plane and train crashes, fires and industrial accidents); and (3) complex emergencies (when the effects of a series of events or factors prevent a community from accessing their basic needs, such as water, food, shelter, security or health care) [1, 3, 8]. Hence, complex emergencies are typically characterised by extensive violence and loss of lives (case fatality); displacement of populations; widespread damage to societies and economies; the need for large-scale, multi-faceted humanitarian assistance; and the hindrance or prevention of humanitarian assistance by political and military constraints and significant security risks for humanitarian relief workers in some areas. Although individuals can be diagnosed definitively in a laboratory through blood tests, collecting samples from patients is an extreme biohazard risk with testing conducted under maximum biological containment conditions [7]. Severely ill patients require intensive supportive care and intravenous fluids. In the ongoing Ebola outbreak, the increasing geographical distribution in the region and the rising fatality rate is due to a lack of specific treatment or vaccine, although new drug therapies are being evaluated.

Implementing the new WHO roadmap synchronised coordination with 3–4 times scaling up for impactful international responses will help the affected countries stop the ongoing transmission. This requires several detailed conventional and non-conventional global responses, estimated to cost at least US $600 million. The military humanitarian involvement announcements for the two countries (Liberia and Sierra Leone) follow a strongly-worded statement issued on September 2^nd^, 2014 by Doctors Without Borders (MSF), which states that world leaders are failing to address the worst-ever Ebola epidemic. It called on states with biological disaster-response capacity both civilian and military to send assets and personnel to West Africa. In a speech to the United Nations, the MSF International President stated that the Ebola epidemic has overstretched the response capacities of West Africa’s health ministries and non-government organisations (NGOs). In the past, MSF has discouraged military interventions in national health emergencies but the ongoing Ebola epidemic transmission has reached levels that can’t be contained without a massive deployment of specialised medical units to boost control efforts [1, 7]. The Ebola tsunami is creating a dire need for more health workers in Liberia, who unfortunately are often unknowingly exposed to patients with a virus with symptoms that mimic other diseases such as malaria, or while working in wards that aren’t designed for EVD treatment. The WHO reported that 152 of Liberia’s health workers have been infected with the disease. A total of 571 health-care workers (HCWs) are known to have been infected with EVD: 93 in Guinea; 332 in Liberia; 2 in Mali; 11 in Nigeria; 128 in Sierra Leone; 1 in Spain; and 4 in the United States of America (2 were infected in the USA, 1 each in Guinea and Sierra Leone). A total of 325 HCWs have died. With the doctor-to-patient ratio already stretched dangerously thin, every infection or death of a doctor or nurse depletes response capacity significantly Following the WHO Ebola Response Roadmap structure, country reports fall into two categories: 1) those with widespread and intense transmission (Guinea, Liberia, and Sierra Leone); and 2) those with or that have had an initial case or cases, or with localized transmission (Mali, Nigeria, Senegal, Spain, and the United States of America). An overview of a separate, unrelated outbreak of EVD in the Democratic Republic of the Congo is also provided. In Mali, there have been so far 4 reported cases, including 4 reported deaths. Whereas, the outbreaks of Ebola Virus Disease (EVD) in Senegal and Nigeria were declared over on 17 October and 19 October 2014, respectively. A national EVD outbreak is considered to be over when 42 days (double the 21-day incubation period of the Ebola [2].

The use of the prime-boost strategy based on the Zaire and Sudan strains attenuated a strain glycoprotein that was first tested on animals for eight weeks. The two-vaccine regimens were effective, though a single dose of the first vaccine provided complete short-term and partial long-term protection. The two-dose regimen protected the chimpanzee adenovirus rather than a human for a full 10 months; earlier tests challenged the macaques only four and five weeks after vaccination, according to the report [7].

## The value of non-conventional interventions against Ebola virus outbreak in West Africa

1. Compassionate use of an experimental drug (serum) in humanitarian crisis

Can an Ebola patient(s) receive ZMapp experimental serum? The news seemed astounding to the scientific community and the affected communities in Africa. It was reported that the two Americans being treated at Emory seemed to have been revived and possibly survived the Ebola infection. In the absence of a drug or vaccine to treat Ebola, the ZMapp experimental serum has been endorsed by the WHO to be deployed on compassionate grounds or benevolence action [7, 9].

In certain situations, the Food and Drug Administration (FDA) allows companies to provide their experimental drugs to people outside of clinical trials, and such use is referred to as compassionate use. But getting access to not yet approved drugs through a compassionate use request can be a long and challenging process. In emergency situations such as an outbreak, access to investigational or experimental drugs for prevention or treatment may be reviewed following an FDA request application and timeline prediction of potential alternative intervention(s). The request must meet some definite criteria, which can include that the company has to comply with the request for an experimental drug or confirm whether the disease is life threatening. Other criteria can include determining if there is availability of any other treatment and if the patient has not been helped by approved treatments. A physician and/or pharmacologist-epidemiologist outbreak expert should also attest with evidence that that the option or benefit of such an experimental drug may pose dangerous unknown risks or be ineffective), on any early preclinical or early clinical study results about the drug trials with documented reports guidance from pharmaceutical industry good manufacturing processes. The Tekmira, Newlink and BioCryst pharmaceutical companies, supported by the US government, the National Institute of Allergy and Infectious Diseases (NIAID), the Department of Defence’s Threat Reduction Agency (DTRA), and the HHS’ Biomedical Advanced Research and Development Authority (BARDA), are working to develop therapeutic candidates for the EVD but are still at the early testing phase (conducting animal studies on safety and toxicity). Hence, it is impossible to discuss or negotiate on the compassionate use of an experimental drug programme for the West African outbreak due to the established scientific clinical trials guidelines and FDA regulations. The need to understand and consult all stakeholders such as human device regulations holders, institutional review boards (IRBs), clinical investigators and FDA staff on humanitarian device exemption (HDE) is highly recommended as new cases are increasing exponentially.

### Medical, ethical and legal considerations of ZMapp experimental serum deployment

In the absence any Ebola drug and/or vaccine, the experimental vaccine was approved by a committee of experts based on compassionate grounds that it might provide protection against the Ebola menace. The WHO announced the endorsement of the implementation of the compassionate use of ZMAPP in providing relief to Ebola patients. The affected populations in West Africa have received the consent of the WHO/AFRO regional, African affected government and international humanitarian organisations in frontline responses and interventions [9, 10]. Over the past nine months, the WHO/AFRO and affected West African governments and stakeholders, including some humanitarian organisations, have consented on the compassionate use and deployment of experimental ZMapp serum, however, it is not certain what the positions are and what reserve of populations is in crisis. Currently, these drugs/vaccines or therapies have shown to be efficacious in animal models. Notwithstanding, the lack of substantial pre-clinical and clinical data, as well as pharmacovigilance information, renders these products inadequate and doubtful to prevent or treat a human patient/subject with the EVD, who has a different exposure history, genetic make-up and environmental factors. A number of candidate vaccines and therapies have been developed and tested in animal models, and some have demonstrated promising results. In view of the urgency and severity of the outbreak, the international community is mobilising to find ways to accelerate the evaluation and use of these compounds. Safety in humans is also unknown, raising the possibility of adverse side effects when administered [7]. The use of these products is demanding and requires intravenous administration and infrastructure, such as cold chain and facilities able to offer good and safe standards of care.

A two-day discussion was held, centring on the potential safety and efficacy evidence associated with the use of Ebola therapies and vaccines in chronic case human subjects, with more than 150 participants representing the fields of research and clinical investigation. Ethics, legal and regulatory concerns, combined with unusual factors, including impoverished infrastructure and health facilities, make it imperative and mandatory to provide all evidence to gather the required scientific, pharmacological and toxicological data on any testing of unapproved experimental products. Detailed and accurate population time-point events, and administered product kinetics and dynamics with interactive clinical data collection in such an impractical field have to be mindful of the short- and long-term effects on the most vulnerable populations. This data is vital and informative to evaluate the potential risks and benefits that can help identify the most potential promising product amidst several therapeutic and vaccine interventions. This should be the focus of in-depth clinical evaluation over space and time [7, 11].

Several ethical, health and legal questions appear difficult to answer such as: Can ZMapp obtain individual or community informed consent (‘Yes, I understand, and I’m still willing to participate’) despite the unknown short- and long-term implications of such an experimental drug? Can someone who is gravely ill and who has never heard of the concept of ‘informed consent’ truly consider the implications of taking a drug like ZMapp? What are the risks of participation and non-participation of domestic and international vulnerable populations? How do we ensure that people know they are participating in trials? Could individuals feel coerced because foreign doctors are the ones asking for consent? Does the patient understand that the drug might not work, or might have very negative side effects down the track? When should physicians use science and experience evidence to guide their actions on human study? Also compounding these dilemmas is the fact that ZMapp has never before been tested on humans.

There are no simple answers to these questions of safety concerns, and the dilemmas faced by scientists and public health workers with respect to using ZMapp are complex. Ethical criteria must guide the provision of such interventions, including transparency about all aspects of care, informed consent, freedom of choice, confidentiality, respect for the person, community preservation of dignity and involvement. The need to prioritize appropriate promising evidence care, fairness on the risks and benefits of using experimental treatments, and timely information dissemination to the community during and post trails as ascribed in Food and Drug Administration (FDA) regulations as well as compassionate recovery/rehabilitation public-health programmes and measures. Except for the Ebola crisis and death toll, any scientific, ethical and justifiable moral reasoning remains unclear. Hence, transparency about all aspects of prevention and care including pharmacovigilance of any affected community in Africa and elsewhere is imperative. The ultimate goal will be doing robust science and research for by minimizing detrimental side effects and better understand how we might tackle the epidemic containment and eradication for the common good of humanity caring services, scientific rationale and effectiveness of potent vaccines and drugs, and least infringement; public justification for social justice and for global prosperity.

Lessons learnt from unethical and illegal tragedies resulting from research travesties include the following: Pfizer’s disastrous trovafloxacin clinical trial during the 1996 meningococcal meningitis outbreak in impoverished settings in Nigeria [12]; the US Public Health Service (PHS) study of untreated syphilis in black Americans (male negro), better known as the Tuskegee Syphilis Study (1932–1972); the US PHS Inoculation Sexually Transmitted Diseases (STD) studies in Guatemala (1946–1948); and the thalidomide tragedy of birth defect epidemics (1950s–1960s) against the Nuffield Council of Bioethics and Universal Declaration of Human Rights, as well the Helsinki Declarations [13, 14]. Understanding the cultural context of risks and benefits, evaluation of the consent and potential presence of undue influence or coercion and cultural sensitivity in the review procedure, the equitability of local enrolment, comprehensive databases of the local population on the actual epidemiology of outbreaks, privacy and confidentiality concerns, and evaluating the long-term welfare of human participants after ZMapp research are imperious and mandatory in line with clinical trial basic requirements and regulations.

It is also worth mentioning that the joint WHO/AFRO, the Economic Community of West African States (ECOWAS) and stakeholder expert committees endorsed the following consensus:

1. The use of whole blood therapies and convalescent blood serums needs to be considered as a matter of priority;
2. Safety studies of the two most advanced vaccines identified based on the vesicular stomatitis virus (VSV-EBO) and chimpanzee adenovirus (ChAd-EBO) are being initiated in the US, and will be started in Africa and Europe in mid-September 2014. The WHO will work with all the relevant stakeholders to accelerate their development and safe use in affected countries. If proven safe, a vaccine could be available in November 2014 for priority use in healthcare workers;
3. In addition to blood therapies and candidate vaccines, the participants discussed the availability and evidence supporting the use of novel therapeutic drugs, including monoclonal antibodies, RNA-based drugs and small antiviral molecules. They also considered the potential use of existing drugs approved for other diseases and conditions. Of the novel products discussed, some have shown great promise in monkey models and have been used in a few Ebola patients (although, in too few cases to permit any conclusion about efficacy);
4. Existing supplies of all experimental medicines are limited. While efforts are underway to accelerate production, supplies will not be sufficient for several months to come. The prospects of having augmented supplies of vaccines rapidly look slightly better;
5. The ‘participants’ cautioned that investigation of these interventions should not detract attention from the implementation of effective clinical care, rigorous infection prevention and control, careful contact tracing and follow-up, and effective risk communication and social mobilisation, all of which are crucial for ending these outbreaks; and
6. The recipients of experimental interventions, locations of studies and study design should be based on the aim of learning as much as we can as fast as we can without compromising patient care or health worker safety, with active participation of local scientists and proper consultation with communities [7].

However, based on the Internal Health Regulations (2005) and the Human Rights Declarations, the use of human subjects in humanitarian crisis situations requires adherence to crucial elements in regards to M&E and all aspects of vaccine/drug pharmacovigilance: (i) appropriate protocols must be rapidly developed for informed consent and safe use; (ii) effective and reliable mechanism for evaluating pre-clinical data should be put in place in order to recommend which interventions should be evaluated as a first priority; (iii) a timely platform must be established for transparent, real-time collection and sharing of data, finally detailed, (iv) consistent regular short- and long-term safety monitoring boards need to be established for these vulnerable populations; and (vi) continuous evaluation of medical, clinical and health data from all interventions with systematic, transparent, liable and responsible short- and long-term exposed population health record assessments, information and community updates, as well as appropriate compensation and rehabilitation programmes, if any.

2. Humanitarian military intervention in the Ebola outbreak emergency response

The most important question concerning this intervention is: can around 3,000 US and UK military forces coupled with local military/policy do and accomplish the tasks at hand, or can they help with relief logistics and implement the much-needed public health programmes?

In the meantime, the demands of alternative responses to the Ebola outbreak have completely outstripped the government’s and relevant partners’ capacity to respond. Fourteen of Liberia’s 15 counties have now reported confirmed cases [2]. The international community has a responsibility to mount a humanitarian intervention by outside forces, and authorise member states to take all necessary measures and change strategy in humanitarian interventions backed by regional or global combined bodies to protect vulnerable populations in West Africa. These relief measures include quiet diplomacy, constructive engagement, provision of relief materials, adequate sheltering and food aid for the hard-to-reach communities using military aircrafts, and confidence building for the more confrontational means of adhering to outbreak emergence guidelines to stop the transmission. In addition to community and national conventional intervention approaches and programmes, the military could be used to reinforce the rule of law in contact tracing or in changing burial cultural practices, and to limit community resistance and hostility against health staff. In a humanitarian military intervention, the violation of nation’s/state’s sovereignty for the purpose of protecting human life should be cautionary. It is important to avoid humanitarian organisations, government/ethnic group preventable repression, famine, civil breakdown and preventable death, as well as guarantee success with varying degrees of seriousness depending on the severity and impact of the Ebola humanitarian crisis coordinated use of mixed conventional and non-conventional emergency responses [15–17].

The concept of national sovereignty has long been the chief legal and political obstacle to military intervention in pursuit of humanitarian objectives, linking respect for human rights with world peace, thereby allowing for the preservation of the principle of sovereignty and non-interference. This principle of sovereignty was established in modern times with the Treaty of Westphalia (1648 exercised by governments on behalf of the people, more or less democratically. Sovereignty thus became the cornerstone of human rights legislation, which brought an end to the Thirty Years’ War and a century of destructive religious conflict in Europe. The benefit of the principle of sovereignty, and its corollary of non-interference in the affairs of another state, was the end to confessional conflicts/wars [15, 17]. The negative result was the growth of absolutist government where sovereignty was located in the person rather than the ruler. African and Asian nations (not nation-states) were invaded and conquered, sometimes in the name of civilisation and humanitarianism. In contrast to the contemporary debates around intervention in Bosnia and even Iraq reflect the same sort of hesitation to intervene when the main issue is a regime’s treatment of its own subjects [18].

For these reasons, consideration of a military humanitarian intervention should be subject to rigorous preconditions, which have rarely, if ever, been met in practice. Military intervention, if acceptable at all, should be a last resort. Where military intervention is contemplated or implemented, there has always been a history of inept or damaging diplomacy and peacekeeping, and inadequate or incompetent relief programmes by the international community. Alternatives, if tried, rarely have been tried properly. In every case in which military intervention has been tried or is contemplated, observers with detailed knowledge of the situation can point to missed opportunities and serious blunders [15, 17].

### Military intervention motivations and humanitarian intervention

The Ebola outbreak has been spreading like wildfire devouring everything in its path across West Africa. Customary international law has always recognised a principle of military intervention on humanitarian grounds. The classic examples of 19^th^-century ‘military humanitarian intervention’ history allow us to take a more sceptical view with regard to the interests at stake. Nonetheless, the theoretical and legal debate has been sophisticated. In the case of the Ebola outbreak, non-intervention – as in the case of a revolution which may sometimes snatch a remedy beyond the reach of law, its essence is legality and its justification – should be at the helm of its success in curbing the Ebola crisis, strengthening the delivery of health care to the far-to-reach and most vulnerable communities, restore hopes and foster sustainable development [19].

For example, European confidence in its ‘civilising mission’ was severely tested by the experience of dictatorship, beginning with the Italian invasion of Ethiopia in 1935. The UN Charter was therefore drawn up in the context of extreme scepticism about ‘humanitarian’ justifications for intervention purposes [18]. Critics of military humanitarian intervention argue that it is no accident that the doctrine of humanitarian intervention in customary law was so abused that it had become worthless. Advocates argue that the UN Charter is designed to restrict the use of force to self-defence and collective action in support of peace and human rights. Over the last 40 years, a number of governments have justified unilateral military action with reference to the customary law of military humanitarian intervention in one form or another. Without exception, the international community has refused to recognise these actions as legitimate. Clear instances are Vietnam’s invasion of Cambodia and Tanzania’s invasions of Uganda, both in 1979. In all these cases, the absence of UN sanction of the military action has been of paramount importance in the wider refusal to condone the actions as true cases of humanitarian intervention [18].

In a globalised world, military humanitarian intervention might have recently undergone a revival in circumstances where national sovereignty has manifestly failed to serve the citizens of a given state. If an abusive government such as the one in Iraq or Sudan cites ‘sovereignty’ to defend actions involving mass violations of human rights (or, in extremis, genocide), then it is clearly failing to exercise that power on behalf of the people to whom it is supposed to be accountable. Democratic endorsement can only be seen as the outcome of a genuine international collective will of the community of nations’ consensus on Universal Declaration of Human Rights and IHR (2005), where the benefits outweigh the consequences. This should not be the outcome of manipulation by one or more powerful countries with foreign policy concerns.

The legal status of military humanitarian intervention, although challenging, may be justifiable in the Ebola crisis and the joint WHO-ECOWAS community is united in demanding such action. The problem is that few, if any, cases of military intervention that cite this doctrine come close to the ideal [3, 8, 15, 17]. In fact, application of humanitarian military intervention in practice in the Ebola crisis in West Africa can take a variety of forms: material assistance (through relief aid); sanctions (coercive, non-military pressure to end abusive practices); and the dispatch of military forces to remedy a human tragedy. Response in the form of material preventive or protection relief is difficult and has seldom proven capable to stop the Ebola outbreak based on ongoing unnoticed and counterproductive efforts of relief organisations with resulting long-term health and economic consequences. Situations of assistance are even more problematic since they are likely to have strategic military significance [3, 17, 20]. For example, the large-scale provision of aid to Ethiopia in the mid-1980s helped to make possible counterinsurgency campaigns that were deeply damaging to the rural poor. In a nutshell, material relief or diplomatic interventions with humanitarian goals not to mention coercive steps such as sanctions – are loaded with strategic significance, may be difficult to implement and are rarely done particularly well due to incompetence or mixed motives by the UN or other representatives of the international community. This is an essential point to grasp before considering the merits and demerits of military intervention in pursuit of genuine and holistic humanitarian aims and responses in Africa.

3. Promoting the use of non-conventional sheltering for Ebola victims and survivors

During the ongoing Ebola outbreak, though households and buildings have been inspected and determined to be safe, several vulnerable populations fled their homes to take refuge in urban cities across borders and elsewhere, with the hope that governments and the international community would provide more befitting security, as well as daily basic medical services for their survival. This requires donations; health worker volunteers; logistical support/anchorage management; public health, medical and mental health services; food services for families; survivors’ reunification to local culture, customs and security of lives and properties [20, 21]. Also there’s need for contingency and emergency evacuation planning and recovery programmes.

Emergency responses, management agencies and jurisdictions are recognising the need to plan for sheltering operations as a result of their historical use following catastrophic events when the capacity of traditional congregate shelters is exceeded. Some examples are the Northridge Earthquake (1994), Hurricane Katrina (2005) and the Samoa Tsunami (2009), just to name a few. Open space shelters or other shelter options need to be appropriate for the local environment and weather conditions (not all tents are designed for all weather conditions) [20].

To capture pertinent information regarding the historical use of non-conventional sheltering, we focused on two sheltering models: (1) mega-shelters, which are large facilities (e.g. stadiums or conference centres) that can house large groups of evacuees, and (2) open space shelters, which are large outdoor environments (e.g. funfair grounds or parks) and use soft-sided or temporarily constructed structures. These require high levels of coordination and organisation between the public and private sector. Spontaneous open space shelters have been initiated for the Ebola-affected population, and other open space shelters have been initiated by governments, NGOs and humanitarian organisations (schools, hospitals/clinics, stadium, recreation centres and parks).

4. Scaling up nutrition and utilisation of non-conventional food aid resources

Although a number of nutritious food resources are both cultivated and gathered in the different ecological zones of Africa, Ebola treatment centres, food security, balanced nutritious diets, hunger and malnutrition remain major challenges across the continent [22]. Reasons why the food and agricultural sector performs poorly in the different geographical parts of Africa are external, internal and natural. With the growing capacity of West African countries to import food to supplement inadequate domestic production and consumption supplies, keeping these food resources for times of crises/disasters should have been kept a priority [8]. Non-conventional food aid resources should be provided for and delivered to vulnerable populations in the respective communities and countries, and timely national agricultural and livestock empowerment response programmes, raising awareness and providing the civilian population with constant information, training and capacity building, and guidance and support, and information management as well as grooming entrepreneurship culture. Motivating, educating, stimulating and persuading the public to employ lifesaving measures or mechanisms that trigger the effectiveness and efficiency of the cultural behaviour and daily life practices and in emergency situations should also be encouraged [23, 24].

The presence of Western relief agencies can give spurious humanitarian credentials to military operations designed to displace and impoverish rural communities. The relief programmes in humanitarian crisis that have been successful have been implemented in concert with attempts to address the strategic context as well and capitalize on local health workers towards optimal mobilization and participation in community interventions. For example, in 1989, Operation Lifeline played a key role in restoring a degree of normality to southern Sudan, devastated by war and famine [23]. There was a simultaneous ceasefire brought about by internal political processes in Sudan. The ceasefire made it possible for rural people to return home, plant crops and herd their animals in confidence that they would not be attacked. Trade and labour migration also became possible. The economic benefit of these activities was far greater than the provision of relief, though the latter received much more international publicity on future outbreak global response responsibilities and actions [25].

How can the strategic context of the famine and malnutrition caused by the Ebola outbreak best be addressed? UN or World Bank resolutions and other diplomatic and economic measures? Unfortunately, such are not given the chance to work, are broken or are only attempted too late. In addition, many conventional interventions actually contribute to varied degrees of human suffering and hardship especially regarding vulnerable populations in developing countries. Thoroughly constructive and participatory diplomacy achieved by associating and guiding local civic groups, notably the churches, and local NGOs to promote alternative support initiatives is equally vital and proving to be productive. The deployment of UN peacekeeping forces can usually be classified as a diplomatic, rather than a military, intervention. Peacekeepers are deployed with the consent of the combatant parties as part of a diplomatic process.

5. Management of non-conventional humanitarian interventions

Management should involve system enhancement and implemented through joint efforts guided by evidence-based information both from community and frontline humanitarian organisations. Timely and fit-for-purpose responses should be instituted in promoting trust, cooperation and prompt recovery through accessible and 24 hour-functioning Ebola healthcare centres and delivery of other public healthcare services nationwide. The need to encourage sustainable mobile health or web-based health application surveillance and early warning alert systems towards rapid information and communication management and tracing is also important. Public and private sector partnerships in emergency management and recovery services of non-conventional interventions require further careful research in filling knowledge gaps and issues. However, most people are aware of the active role any non-profit organisations take in disaster relief; for-profit contractors or organisations/country may not be as obvious but often play just as important a role. However, with the outlay of government support also comes a variety of opinions (e.g. political agenda) which dictate where and how the resources should be spent. Innovative partnerships should be effective, aimed at improving and alleviating burden, and preventing death from the outbreak in the field by utilising new methods and state-of-the-art approaches of active mitigation, collection responsibility in preparation, response and recovery under the provincial, national and regional emergency response framework.

In light of the potential success of the formal and/or informal systems based on trust, confidence and credibility, accountability and distribution systems that enable access to the affected communities to determine what is required and how to proceed – and potentially combining oversight and responsibility with more decision-making power on the ground and increased flexibility – should be developed. The level of creativity allows member organisations to address needs that could not be met through traditional government channels, perfectly illustrating how the work of NGOs and the governments they are associated with should move forward hand in hand. Of course, for all the positives that come with a more active public, there are some difficulties associated with an increased level of local and private-sector involvement. Inherently connected to a dispersion of authority comes a lack of standardisation in the quality of work, and differing views on the appropriateness or effectiveness of any given practice. However, a rapidly developing and stretched emergency can also bring these humanitarian service providers fatigue to the point of breakdown.

As evidence of the growing philosophy of ECOWAS’ cooperation, volunteer organisations in emergency response and management are generous with their time, money and community outreach programmes, and coordinate planning efforts that can impact significantly on communities. Active and open relationships between governments and non-profits or businesses with clear guidelines for emergency chain procurement are crucial: without regular testing and practice, all the arrangements and memoranda of understanding are worthless. Excessive rigidity in a system designed to deal with the chaos of an emergency is a recipe for failure as trying to prevent past mistakes brings about many questions about how future pandemics will actually be prevented [1, 7]. The key in moving forward and continuously improving our abilities is to push for a greater sense of cooperation and synchronised coordination between the public and private sectors, including sharing of expertise and resources in globalisation. There are many parts of the field that need work and with limited resources, it can often be difficult to accomplish everything with innovative solutions and leadership in order to overcome the challenges currently faced and improving our technical and structural processes. One of the crucial adjustments must be an honest assessment and comprehensive approach of current funding arrangements, especially in light of the fact that important programmes are not always the ones that get flashy media attention, as well as impact funding opportunities that encourage communities to take control of their own preparedness. For example, people prefer to have a say in what happens to them, and the coordination of efforts between the government and the public offers a sense of control over situations that can be exceedingly chaotic. All in all, there are currently glimpses of how safe and secure we can make our country and the entire regional sustainable development.

There is an urgent need for concrete measures to reduce the vulnerability of societies to deal with outbreaks, the loss of human lives, and the heavy physical and economic damage that occurs as a result. The development and implementation of joint administrative, technological and scientific approaches, funding mechanisms and policies should be initiated and endorsed by African local institutions and countries including intergovernmental, regional organisations and associations that have adopted, action programmes with the participation of private companies and individuals (including allocation of budgetary resources and the exchange of data and technology, as well as. Importantly, the WHO roadmap guidelines on crisis/disaster prevention, preparedness and mitigation, and its plan of action against the Ebola outbreak could guide priorities commitment and work plan timeline. The development of country initiatives to preparedness and disease surveillance response promotion, enhanced exchange and stronger regional cohesion, M & E translation into concrete activities and actions in close cooperation with all stakeholders comprising the affected communities, governments and international framework of actions. Nurturing timely mobilisation of domestic resources for sustained financial and structural stability and establishment of functioning early warning and surveillance response systems is invaluable evidence of effective and concrete programmes and activities implemented based on local context and in strengthening existing health system. Similarly, commitment and funding to study conventional and non-conventional measures and effective programme priorities and guidance, both at the national level and with respect to sub-regional, regional and international technical cooperation, are essential [3, 7].

Conclusively, for successful outbreak reduction and containment programmes, necessary support should be provided to national and regional policy and to global strategy development, public awareness building and resource mobilisation should be fostered networking.

The scientific community should be encouraged and national committees should be supported to integrate transdisciplinary and cross-sectorial programmes at all levels. Effective and efficient, accurate and timely outbreaks early warning capacities, prevention, preparedness measures and strategic dissemination of information capitalising on advances in telecommunications such as social media, internet and broadcast services are key factors to successful crisis/disaster prevention. The need to mitigate on improvements in coordinating platforms and its plan of action, and capacity building in community/national programmes and activities, is crucial in promoting reliable and robust emergency relief systems and monitoring adverse impacts with contingency plans for sustainable development.

## An uncertain future

Beyond operational and political concerns, humanitarian military intervention also involves legal issues outside of the UN Security Council mandate. In non-conventional interventions, accountability and human rights must be respected and could be part of an innovative model for emergency response to global disease outbreaks based on lessons learnt from peace and conflict resolutions from previous military intervention outrages in conflict events worldwide. The willingness to use non-conventional interventions is inevitably influenced not only by the desperation of the affected population and cancellations of commercial flights in West Africa, but also by economic and geopolitical factors, including the relevance of the country to the world community and its regional stability. The attitudes of other major global health players have prompted discussions on the impact of commitment and prompt actions on Ebola outbreak humanitarian crisis in West Africa and the urgency for African countries to embark on proactive planning and steps in emerging disease pandemic preparedness, early warning indications, surveillance and emergency response. As both top-down protection and bottom-up empowerment systems should be comprehensive, multi-dimensional, necessary to realise human security and protection from disease outbreak and limit crises, monitoring goals and indicators should not be limited to prevention and protection measures, but should also include perspectives for risk reduction management, early warning and strengthening of resilience. Strengthening of socioeconomic and environmental capital is an important component for community capacity building and empowerment towards entrepreneurship and community ownership of health programmes and interventions, as well as service delivery, thus contributing directly to identifying and implementing solutions, and individual and societal capabilities to measure and monitor their performance. How can predictions mimic naturally both animal and human occurring disease outbreaks, lives and economic upheaval from future mystery illnesses with quantified contemporary uncertainty, understanding of force-of-infection and duration threatening to increase in the near future?

## Conclusion

Solving global health problems are increasingly becoming too complicated to be addressed by single actors, multi-sector actors and approaches rather than “one humanitarian model fits all”, comprehensive population’s centered and context-specific approaches. Global community and human security, which all highlight concerns with various threats and perils such as wars, violent conflicts, natural disasters, catastrophic accidents and illness, should be given close attention to enhance its preparedness and emergency response platforms. It is unrealistic and inefficient to expect each country to be prepared for potential threats: strategic international partnership is required to collaboratively share the risks and strengthen societal resilience towards sudden outbreaks/shocks. Regional cooperation and global cooperation have to be developed to enhance preparedness to deal with large-scale hazards and mitigate sustainability, protection and empowerment, and recovery and rehabilitation programmes based on the best and most robust scientific information and coordinated public programmes in urban and rural areas.

## Electronic supplementary material

Additional file 1: **Multilingual abstracts in the six official working languages of the United Nations.** (PDF 275 KB)
