# Supplementary material for: Non-conventional humanitarian interventions on Ebola outbreak crisis in West Africa: health, ethics and legal implications
Source: Infect Dis Poverty. 2014 Nov 25;3:42. doi: 10.1186/2049-9957-3-42 (PMC4323207; doi:10.1186/2049-9957-3-42)
Supplement: Supplementary file 1 — Additional file 1: Multilingual abstracts in the six official working languages of the United Nations. (PDF 275 KB) [file 40249_2014_82_MOESM1_ESM.pdf]

Translation of the abstract into the six official working languages of the United Nations

التدخل الإنساني غير التقليدي
في أزمة تفشي مرض الإيبولا في منطقة غرب إفريقيا: الصحة والأخلاق والآثار القانونية

إي تامبو

الملخص

نظرا لغياب أنظمة الإنذار المبكر، وأنظمة المراقبة والإستجابة فقد تفشي المرض الأكثر فتكاً، والأعقد، والأوسع إنتشاراً وهو الإيبولا، المرض الذي دمر مجتمعات غرب إفريقيا. وقد دفع تسونامي مرض الإيبولا بتساؤلات عديدة، وإستجوابات، بل وشكوك كثيرة حول مدى فعالية وكفاءة إستعدادات المجتمعات الوطنية والإقليمية والدولية لنشر وسائل السيطرة التقليدية في حالات الإنتشار المماثلة من خلال المنظمات الإنسانية والهيئات المحلية غير الحكومية للحد من شدة حركة إنتقال المرض والعمل على وقف تفشيه المُستمر. وقد بلغت حالات الإصابة بالمرض إلى عدد غير مسبوق من الإصابات وصل إلى7429 حالة، منها 3439 حالة وفاة وذلك إعتباراً من بداية شهر أكتوبر 2014 كنتيجة للقضايا والتحديات التي تواجه تقديم المساعدات الإنسانية للحد من تفشي مرض الإيبولا وجودة مكافحته في منطقة غرب إفريقيا. كما ساهمت عوامل مُتعددة في تفاقم أزمة مرض الإيبولا، نذكر منها على سبيل المثال أنظمة المجتمع الهشة، والأنظمة الصحية الوطنية الضعيفة، وعدم كفاية الموارد، وسوء الطرق، وأنظمة النقل، وسكن العشوائيات، والمرافق الإجتماعية القاصرة، وسوء الحكم في المنطقة، والصراعات/الحروب والإضطرابات السياسية التي ضاعفت من حدة الأزمة. علماً بأن القارة الإفريقية تمتلك نسبة كبيرة من ثروات العالم، ولكن سكان القارة الأفارقة يعيشون في فقر ذليل وحقير، في ظل حكومات غير قادرة على توفير الطعام والحكم بشكل فعال، حيث حُكم عليهم بتحمل لحظات حالكة السواد تتزامن مع تفشي مرض الإيبولا في غرب إفريقيا. وعليه، ولذا توجد حاجة ماسة إلى إتباع نهج علمي أكثر واقعية وقوة لتحويل ولإعادة توجيه الموارد الطبيعية الهائلة والإمكانات البشرية المتوفرة من أجل تحقيق التغطية الشاملة وتطوير الأهداف نحو تحقيق أهداف التنمية المستدامة في المنطقة، وتطوير المنطقة وفق الأهداف التنموية للألفية 2015-2030. كما أن التنظيم المؤسساتي للنظم الصحية الفعالة وطرق الإستجابة غير التقليدية لحالات الطوارئ مثل توفير الخدمات والإعاشة الحياتية (كتقديم الخدمات الصحية الطارئة، والمساعدات الغذائية، والملاجئ الطارئة، والمواد الواقية، والمياه والكهرباء، وتوزيع إمدادات الطوارئ) هو مسؤولية مباشرة تقع على عاتق الحكومات، ومن الممكن أن تُساعد بشكل ملموس لإحتواء الأزمة وبالتالي وقف إنتشار مرض الإيبولا جغرافيا. بالإضافة إلى إمكانية تصميم برامج الإدارة العملية والتشغيلية المكون من عدة مستويات، وإعداد نماذج للمساعدة في إدارة الأزمات الشابهة لأزمة تفشي مرض الإيبولا. فضلا عن ذلك، العمل على توفير خدمات الصحة العقلية الجماعية في حالات الطوارئ لعلاج الأعدادالضخمة ممن يُعانون من الآثار الصحية والإجتماعية والنفسية والإقتصادية في الفترات التالية للأزمات.

Translated from English version into Arabic by FREELANCEABUNAR, through

TRANSLATORS WITHOUT BORDERS

西非埃博拉暴发危机中的非传统人道干预：健康、伦理与法律意义

E Tambo

摘要

由于没有埃博拉暴发早期预警预报、监测和响应体系，致死性最强、最复杂和最大的埃博拉疫情已在摧毁西非社区。空前的埃博拉疫情促使人们质疑国家、地区和国际社会通过人道主义和地方非政府组织准备配置控制传播并阻止疾病持续扩散的传统疾病暴发防控措施的效率。西非埃博拉暴发中人道援助存在的问题与挑战到2014年10月初已经导致7,429例病患，其中3,439例死亡病例。脆弱的社区与国家卫生体系，资源匮乏，公路与交通不便，贫民窟，公共设施短缺，地区政府治理不力，冲突和战争以及政治动乱等都使疫情更加恶化。非洲大陆拥有世界上大部分的财富，然而她的人民生活在赤贫，政府无法养活和有效地管理他们，以致他们不得不忍受埃博拉疫情这种比以往更加黑暗的时刻。解决这些问题关键需要一个更加务实和稳健的科学方法向着实现全民覆盖和发展2015-2030年发展目标(MDGs)以及向着本地区的可持续发展进行自然和人力资源潜能的转变与重新调整。如果制度化的高效卫生系统和诸如提供维持生命和生物防护等服务（紧急医疗服务，食品援助，紧急避难和防护材料，水和电，以及应急物资分配）的非传统应急响应方法能有效落实，那无疑将有利于控制并最终阻止埃博拉的进一步扩散。实用并可操作的管理级项目与模型的目的就是能应对像埃博拉疫情一样的危机。除此之外，紧急大规模精神卫生服务是处理灾后大规模健康、社会心理和经济损失等问题所必不可少的。

Translated from English version into Chinese by Yin Jian-hai, through

CDC CHINESE CENTER FOR DISEASE CONTROL AND PREVENTION
NATIONAL INSTITUTE OF PARASITIC DISEASES

Intervention humanitaire non conventionnelle dans la crise de l'épidémie d'Ebola en Afrique de l'Ouest : implications sanitaires, éthiques et juridiques

E. Tambo

Résumé
En raison de l'absence de systèmes d'alerte précoce, de surveillance et d'intervention pour l'épidémie d'Ebola, la guerre contre Ebola, la plus meurtrière, la plus complexe et la plus importante jamais vue, a dévasté les communautés d'Afrique de l'Ouest. Un tsunami Ebola sans précédent a suscité des interrogations et des incertitudes quant à l'efficacité et l'efficience des communautés nationales, régionales et internationales, et leur capacité à déployer des méthodes de lutte classiques contre les épidémies par l'intermédiaire d'organisations humanitaires et non gouvernementales locales pour en freiner la dynamique de transmission et en arrêter la propagation. Les problèmes et les défis de l'aide humanitaire dans l'épidémie de fièvre Ebola en Afrique de l'Ouest ont conduit à un bilan sans précédent : 7 429 cas et 3 439 décès, début octobre 2014. La fragilité et la faiblesse des systèmes de santé communautaires et nationaux, l'insuffisance des ressources, le mauvais état des routes et des systèmes de transport, les bidonvilles, les déficiences des institutions sociales, la mauvaise gouvernance régionale, les conflits, les guerres et les troubles politiques mettent de l'huile sur le feu Ebola. Le continent héberge une forte proportion des richesses du monde, et pourtant ses habitants vivent dans un état de pauvreté abjecte, les gouvernements sont incapables de les nourrir et de les gouverner efficacement, et ils sont condamnés à endurer des moments encore plus sombres avec l'épidémie d'Ebola en Afrique de l'Ouest. Nous sommes devant un besoin critique d'une approche scientifique plus pragmatique et plus robuste, consistant à transformer et à réorienter les énormes potentialités en ressources naturelles et humaines pour atteindre une couverture universelle et les Objectifs du Millénaire pour le Développement (OMD) 2015-2030, d'une croissance et d'un développement durables dans la région. L'institutionnalisation de systèmes de santé efficaces et de méthodes d'intervention d'urgence non conventionnelles, telles que la fourniture de services vitaux et biodéfensifs (prestation de services de soins de santé d'urgence, aide alimentaire, abris et matériel de protection d'urgence, eau et électricité, distribution de fournitures d'urgence) tombent en droite ligne et peuvent clairement contribuer à contenir et finalement arrêter la géo-distribution d'Ebola. Des programmes et des modèles de gestion pratiques et opérationnels peuvent être conçus pour aider à gérer des crises comme les épidémies d'Ebola. En outre, des services d'urgence de santé mentale de masse sont nécessaires pour traiter l'énorme variété des conséquences sanitaires, socio-psychologiques et économiques consécutives aux crises.

Translated from English version into French by Jacek Sierakowski, through

TRANSLATORS
WITHOUT BORDERS

Нетрадиционное гуманитарное вмешательство в очаге заболевания лихорадки Эбола в Западной Африке: последствия для здоровья, а также этические и правовые последствия.

Е Тамбо

Синопсис
Вследствие отсутствия систем раннего предупреждения, наблюдения и реагирования очагов возникновения лихорадки Эбола, самая сложная и смертоносная в истории континента война с заболеванием охватила населенные пункты западной Африки. Беспрецедентная волна распространения заболевания заставила задуматься о том, насколько эффективны традиционные средства реагирования и контроля распространения заболеваний на национальном, региональном и международном уровне, предоставляемые гуманитарными и местными неправительственными организациями. Проблемы, стоящие перед организациями, оказывающими гуманитарную помощь во время вспышки лихорадки Эбола в западной Африке, привели к беспрецедентному количеству зарегестрированных случаев заражения – 7429 и 3439 летальных исходов к началу октября 2014 года. Слабые и неподготовленные местные и государственные системы здравоохранения, отсутствие дорог и транспортных систем, трущобные условия жизни, недостаток общественных институтов, слабые органы управления на местах, военные конфликты и политические дрязги способствовали распространению лихорадки Эбола. На континенте сконцентрирована большая часть мировых ресурсов, однако население живет в ужасающей нищете и правительства не способны создать эффективные условия управления и распределения ресурсов. Условия жизни населения западной Африки безусловно ждет дальнейшее ухудшение, вызванное Эболой. Для достижения Целей Развития Тысячелетия 2015-2030 (Millennium Developing Goals (MDGs) необходимо более прагматично и с использованием строго научного подхода кардинально изменить и

переориентировать огромный потенциал естественных и человеческих ресурсов в направлении устойчивого роста и развития региона. Организация эффективных систем здравоохранения и нетрадиционные методы реагирования в чрезвычайных ситуациях, такие как предоставление экстренных услуг поддержания жизни и биозащиты, а именно услуги скорой помощи, распределения продовольственной помощи, сооружения приютов и защитных материалов, воды и электроэнергии, распределения предметов первой необходимости, являются именно теми мерами, которые остановят глобальное распространение вируса. Возможно сформулировать практические и оперативные программы управления и модели для управления кризисными ситуациями типа вспышек лихорадки Эбола. В дополнение, необходимы услуги экстренной психологической помощи для борьбы с общественно-психологическими и экономическими последствиями этого кризиса.

Translated from English version into Russian by Julia Zarubinska-Toepritz, through

TRANSLATORS
WITHOUT BORDERS

Intervención humanitaria no convencional en la crisis por el brote del ébola[1] en África Occidental: la salud, la ética y las implicaciones legales

E Tambo

Resumen
La lucha contra el ébola, en las comunidades de África occidental, ha sido la guerra más grande, más mortal y devastadora que se haya visto y esto debido a la ausencia de un sistema de alerta precoz, de vigilancia y de respuesta frente al brote. El tsunami, sin precedentes, del ébola ha provocado interrogantes e incertidumbre acerca de la efectividad y eficiencia de la pericia de las comunidades tanto nacionales, regionales e internacionales para implementar mecanismos de control convencionales a través de organizaciones no gubernamentales locales y humanitarias para frenar la dinámica de transmisión y la propagación imperante. Los problemas y desafíos que enfrenta la ayuda humanitaria por el brote del ébola en África occidental, están encabezados por un número sin precedentes, hasta principios de octubre de 2014, de 7.429 enfermos y 3.439 muertos. Los sistemas de salud comunitarios y nacionales deficientes y frágiles, los recursos inadecuados, los malos sistemas de transporte y carreteras, las viviendas precarias, los servicios sociales deficientes, la mala gobernanza en la región; las guerras/conflictos y la convulsión política, echan más leña al fuego del ébola. El continente reúne una gran proporción de la riqueza mundial y, sin embargo, en África occidental, su población vive en la pobreza extrema, con gobiernos que no pueden alimentarlos ni gobernarlos de manera efectiva y están condenados a soportar momentos más oscuros aún, con el brote del ébola. Es imperioso un enfoque científico más pragmático y firme para reorientar y transformar las enormes potencialidades en recursos naturales y humanos y así lograr una cobertura universal y los Objetivos de Desarrollo del Milenio (ODM 2015-2030), hacia lograr un crecimiento sostenible y desarrollo en la región. La institucionalización de sistemas de salud eficientes y de métodos no convencionales en caso de emergencias, tales como, el soporte vital básico y programas de defensa biológica (la prestación de servicios de atención médica de emergencia, ayuda alimentaria, refugios de emergencia y materiales protectores; agua y electricidad y la distribución de suministros de emergencia), incumben llanamente y pueden ayudar a refrenar y, en definitiva, detener la expansión del ébola en el territorio. Pueden diseñarse programas y modelos a nivel de gestión operativa para colaborar en el manejo de crisis, como es el caso del brote del ébola. Además de eso, es requisito el servicio de salud mental de emergencia masivo, para abordar las grandes consecuencias en la salud, socio-psicológicas y económicas post crisis.

Translated from English version into Spanish by Andrea Pisera, through

TRANSLATORS
WITHOUT BORDERS

[1] Aclaración del uso de la palabra Ébola: http://www.fundeu.es/recomendacion/ebola-escritura-adecuada/
